# Risky sexual behavior and associated factors among patients with bipolar disorders in Ethiopia

**DOI:** 10.1186/s12888-019-2313-2

**Published:** 2019-10-25

**Authors:** Chalachew Shambel Obo, Lamesa Melese Sori, Tadesse Melaku Abegaz, Bizuneh Tesfaye Molla

**Affiliations:** 1 Department of Psychiatry, University of Gondar Hospital, Gondar, Ethiopia; 2 University of South Australia, School of Pharmacy and Medical Sciences, Adelaide, Australia; 3 Department of Psychiatry, University of Gondar, College of Medicine and Health Sciences, Gondar, Ethiopia

**Keywords:** Risky sexual behavior, Bipolar disorder, AMHH outpatient department

## Abstract

**Background:**

People with bipolar disorder are highly vulnerable to risky sexual behaviors (RSBs). The magnitude of RSBs among bipolar disorders was not studied in our population. The present study aimed to explore the prevalence of RSBs and associated factors among patients with bipolar disorder.

**Method:**

An institution based cross-sectional study was conducted from 1 April to 30 May 2017 among people living with bipolar disorder at outpatient departments of Amanuel Mental Health Hospital, Addis Ababa. Systematic random sampling was used to select participants. Risky sexual behavior was defined as having sex with two or more sexual partners, having unprotected sexual intercourse, sex after alcohol consumption, exchanged money for sex in a previous 12 months. Data collection was conducted through face-to-face interview by a structured questionnaire adopted from behavioral surveillance survey. Binary logistic regression was conducted to identify factors associated with RSBs.

**Result:**

A total of 424 participants were enrolled in the study, giving overall response rate of 96%. About 223(52.6%) were males. The prevalence of risky sexual behavior was 49.1% among bipolar patients. Male patients (Adjusted Odds Ratio (AOR) =2.23,95% CI = 1.27,3.92), patients in age group of 18–24(AOR = 2.08,95% CI = 1.47,3.81),current manic phase of the illness (AOR = 2.3195% CI,1.24,4.32) and current alcohol drinking (AOR = 3.70,95% CI = 2.01,6.78) had significant association with RSB.

**Conclusion:**

Almost half of bipolar patients reported a risky sexual behavior. Current manic episode and the consumption of alcohol were independently associated with RSB. To reduce the burden of RSBs, mental health services which focuses on sexual behaviors of bipolar patients is required.

## Background

Risky sexual behavior (RSB) is commonly defined as behavior that increases the risk of contracting sexually transmitted infections and experiencing unintended pregnancies [1]. They include having sex at an early age, having multiple sexual partners, having sex while under the influence of alcohol or drugs, and unprotected sexual behaviors [2]. Usually RSBs are considered as a sexual behavior that increases the chance of negative social impact. These impacts include family conflicts, damage to relationships, legal disputes and even financial problems. Overall definition of RSB is in close relation with dynamic transmission of sexually transmitted infections (STI) due to unsafe sex [3–5].

Being the sixth leading disability in the world, bipolar disorder (BD) is a severe mood disorder characterized by cyclical shifts in mood, alternating between mania and depression [6]. One of the defining symptoms of mania is the tendency to engage in pleasurable activities without regard to potentially dangerous consequences [7]. The constellation of manic symptoms, including euphoria, grandiosity, hyper sexuality, impulsivity, and poor judgment often results in risky behaviors. Because of symptoms like hyper sexuality and impulsivity people with bipolar disorder are highly engaged in RSBs [8, 9]**.** A comparative study conducted in United States of America (USA) demonstrated that patients with mood disorder reported more frequent sexual intercourse, higher frequencies of unprotected sex, a higher history of sexually transmitted diseases and more sexual partners [10]. A large cross-sectional study conducted in Brazil reported that over 90% of bipolar patients reported life time unprotected sex, 30% had exchanged money for sex, and 26.8% had more than 10 sexual partners [11]. Another study from Nigeria reported that (45.7%) of the adolescents with BD had positive history of risky behavior [12]. Local available data revealed that, high prevalence of RSB have been documented in high school, college students and street people in Ethiopia [13–16]. However, there are no figures for prevalence of RSB among bipolar patients in Ethiopia.

Some factors have been implicated with RSB of such as, being single, low economic status, low educational back ground, unemployment, HIV infection and life time history of homelessness were associated with high rate of RSB [11, 17–19]. In addition early onset of illness and previous history of hospitalization were significantly associated with risky sexual behavior among bipolar patients [12]. Behavioral factors such as alcohol consumption and cigarette smoking are commonly reported risk factor due in part to disinhibition, increased libido and impairing judgment [11, 20, 21]. Estimation of RSBs on bipolar patients would help to develop risk reduction strategy for those individuals who are suffering from the problem. Therefore, this study is aimed at assessing prevalence and factors associated with RSB among patients diagnosed with bipolar disorders.

## Methods

### Study setting and design

Institution based cross sectional study was conducted from 1 April to 30 May 2017 at outpatient departments of Amanuel Mental Health Hospital (AMHH) in Addis Ababa, Ethiopia. The hospital is the only specialized and largest mental hospital in the country. It provides inpatient, outpatient and emergency services for many mentally ill people and also contributes a lot as a center of clinical practice for medical and health science students coming from different universities and colleges.

### Source and study population

All patients who were clinically diagnosed with bipolar disorder were our source population whereas patients who attended an outpatient department of AMHH at the time of data collection were our study subjects.

### Inclusion and exclusion criteria

Patients who were 18 years and above with the diagnosis of bipolar disorder were included. We did not include patients who were unable to respond because of severe form of the bipolar disorder such as aggressiveness and agitation. Patients with medical comorbidities that necessitate them for inpatient admission were excluded.

### Sample size determination

The minimum number of samples required for this study was determined by using single population proportion formula considering the following assumption. Accordingly, we included 442 participants assuming 15% non-response rate.

### Sampling techniques

A systematic random sampling technique was used to select the study subjects. The average monthly flow of bipolar patients at out-patients at AMHH by the year 2016 was 926. Sampling interval was determined by dividing the total study population who had a follow up during a month of data collection period (926) by the total sample size (442). Then, every two people were chosen and interviewed by data collectors after patients are evaluated by their doctors.

### Study variables

Our dependent variable was RSB whereas the independent variables include sociodemographic factors including age, gender**,** marital status**,** educational status**,** occupational status, place of residence**,** monthly income and clinical factors such as duration of illness**,** number of disease episodes**,** type of current episode and number of hospitalizations. Furthermore, behavioral factors such as alcohol consumption, cigarette smoking and khat chewing were our independent variables.

### Operational definition

Risky sexual behavior: it was defined as having sex with two or more sexual partners, having unprotected sexual intercourse, sex after alcohol consumption, exchanged money for sex in a previous 12 months. Those who reported behaving at least one aspect of the definition were considered to have had risky sexual behavior [2].

### Cigarette and substance use (CSU)

Substance use: it refers to substances that are highly utilized in Ethiopian population mainly alcohol and khat.

Khat: *Catha edulis* is an evergreen stimulant tree or shrub mainly cultivated in east Africa including Ethiopia.

Life time users: participants who had used substances at least once in their lifetime.

Current users: participants who had reported use of substances at least once in the last three months.

#### Data collection and instruments

##### Instruments

Data collection was conducted through face-to-face interview by using a structured questionnaires that contained risk related sexual information including number of sexual partners, history of unprotected sex, exchanging money for sex, sexual intercourse after using alcohol or other drugs. The questionnaire was adopted from Behavioral surveillance survey and other published articles which was modified to be applicable for the local context [12, 13]. Socio demographic information like age, sex, marital status, religion, employment, place of residence and living situations were asked by using socio demographic questionnaire. Clinical variables such as number of episode, current episode, and age of onset of illness was asked and reviewed from the patients’ clinical notes.

##### Data collection

The face-to face interview was carried out by five trained psychiatric nurses and two psychiatric professionals through interviewer-administered questionnaires and patient chart review was made to gather additional patient information. During the interview**,** the diagnosis of RSB was made upon the reporting of one of the following scenarios over a 12-month period: particularly, having sex with two or more sexual partners, having unprotected sexual intercourse, sex after alcohol consumption, exchanged money for sex.

##### Data quality assurance

One day training was given for data collectors and supervisors on the purpose of the study, details of the questionnaire, interviewing techniques, importance of privacy, and on insuring confidentiality of the respondents. The questionnaire was translated in to Amharic and back translated into English by experienced professionals to check its consistency. Consensus on the compatibility between forward translations was assured before any data collection activities. Before starting the actual survey, the questionnaire was pre-tested on 5% of sample size at a Saint Paul hospital and it was not included in the main analysis. Throughout the course of the data collection, interviewers were supervised at each outpatient department. Regular meeting was held between the data collectors and the principal investigator together in case of any difficulties. At the end of every data collection, the questionnaire was reviewed and checked for completeness, accuracy and consistency by supervisors and investigator to take immediate corrective measures.

### Data processing and analysis

The collected data was coded, entered in to EPI-DATA version 3.1 and transported to SPSS version 20.0 (SPSS Inc. Chicago, IL) software for windows. Descriptive statistics was used to analyses categorical variables. Bivariate analysis was performed to determine each of explanatory variables. Binary logistic regression analysis was done to identify associated factors between RSB and the explanatory variables. The strength of the association was presented by odds ratio and 95% confidence interval. Finally, data was presented by using numbers, frequencies, tables and figures. p- Value less than 0.05 was considered as statistically significant.

## Results

### Socio demographic characteristics of participants

A total of 424 participants were involved in the study with an overall response rate of 95.92%. About 223(52.6%) were males. Approximately one-third of them 136(32.1%) were currently unemployed and 303(71.5%) currently reside in urban areas (Table 1).

**Table 1:** Sociodemographic characteristics of bipolar patients attending AMHH, 2017

Variables(*N* = 424)	Categories	N (%)
Sex	Male	223 (52.6)
	Female	201 (47.4)
Age	18–24	78 (18.4)
	25–34	180 (42.5)
	35–44	134 (31.6)
	> 44	32 (7.5)
Religion	Orthodox	200 (47.2)
	Muslim	132 (31.1)
	Protestant	78 (18.4)
	Others	14 (3.3)
Marital status	Single	227 (53.6)
	Married	128 (30.2)
	Divorced and widowed	69 (16.2)
Educational status	Can’t read and write	21 (5.0)
	Primary	88 (20.8)
	Secondary	166 (39.2)
	College and above	149 (35.1)
Occupation	Gov. employee	75 (17.7)
	Private	121 (28.5)
	Daily laborer	18 (4.2)
	Unemployed	136 (32.1)
	Student	74 (17.5)
Place of residence	Urban	303 (71.5)
	Rural	121 (28.5)

### Clinical and psychosocial characteristics of participants

Majority 225(59.2%) of patients were diagnosed for bipolar disorder before the age of 25 years. Around one-half of the subjects 210(49.5%) had the illness for 5–10 years. About 211(49.8%) of respondents were on treatment for 5–10 years. Three-fourth 318(75.0%) of the bipolar patients had more than two episodes. More than half of 219(51.7%) of the respondents had previous history of hospitalization because of bipolar disorder (Table 2).

**Table 2:** Clinical and psychosocial characteristics of bipolar disorder patients attending AMHH, 2017

Variables	Categories	N(%)
Age at onset of the illness	≤25 years	225 (59.2)
	> 25 years	173 (40.8)
Duration of the illness	≤5 years	119 (28.1)
	5–10 years	210 (49.5)
	> 10 years	95 (22.4)
Duration on treatment	≤5 years	123 (29.0)
	6–10 years	210 (49.5)
	> 10 years	91 (21.5)
Number of episodes	≤2	104 (24.5)
	> 2	320 (75.5)
Type of current episode	Manic	240 (56.6)
	Depressive	184 (43.4)
Presence of hospitalization	Yes	205 (48.3)
	No	219 (51.7)
Number of hospitalization(*N* = 205)	≤4	153 (74.6)
	> 4	52 (35.4)
Life time history of homelessness	Yes	94 (22.2)
	No	330 (77.8)
History of childhood sexual abuse	Yes	12 (2.8)
	No	412 (97.2)

### Substance use related history of the participants

Among 424 subjects involved in this study the majority, 261(61.6%) had used alcohol while 147 (34.7%) reported Khat chewing and 90 (21.2%) of them reported cigarette smoking at least once in their life, respectively (Table 3). Significant number of patients continued consumption of alcohol after diagnosis of the disorder 170(40.1%). However, use of Khat 70(16.5%) and cigarette smoking 56(13.2) declined meanwhile after the diagnosis of bipolar disorder (Table 3).

**Table 3:** Substances use history among people with bipolar disorder at AMHH, 2017

Characteristics	N(%)
Previous substance history	
Alcohol	
Yes	261 (61.6)
No	1639 (38.4)
Khat	
yes	147 (34.7)
no	277 (65.3)
Cigarette	
Yes	90 (21.2)
No	334 (78.8)
Current substance use	
Alcohol use	
Yes	170 (40.1)
No	254 (59.9)
Khat	
Yes	70 (16.5)
No	354 (83.5)
Cigarette	
Yes	56 (13.2)
No	368 (86.8)

### Prevalence of risky sexual behavior among people with bipolar disorder

Out of the total respondents, 371(87.5%) had sexual experience ever. Of the sexually active patients, 192(51.75%) were males. Regarding the ongoing sexual behaviors of participants, 297(79.5%) had sex in the previous 12 months. Of these recently sexually active patients, 147, 49.5%,[95% CI, 43.8–55.6] had involved in at least one form of RSB.

### Factors associated with risky sexual behaviors among people with bipolar disorder

For each independent variable, bivariate analysis was performed. Sex, age group, age at onset of the illness, type of current episode, current alcohol use, current Khat use were variables which had a bivariate association with risky sexual behavior. Accordingly, binary logistic regression analysis was conducted for those variables that were having bivariate association. After adjustment of confounding factors, the binary logistic regression analysis indicated that four factors were independently associated with risky sexual behavior. Accordingly, male gender was found to independently increase the odds of RSB more than two-fold. Male patients had a twofold risk of engaging in RSBs than females adjusted odds ratio (AOR) [2.23, 95% CI = 1.27, 3.92]. Patients in age group 18–24 were 2 times more likely to show RSB, AOR [2.08, 95% CI = 1.47, 3.81]. Current manic episode increased the tendency of RSB more than two times, AOR [2.31, 95% CI = 1.24, 4.32]. In addition, current use of alcohol was found to be associated with high incidence of RSB, [AOR = 3.70, 95% CI = 2.01, 6.78] (Table 4).

**Table 4:** Binary logistic regression analysis of factors associated with RSB among people with bipolar disorder at AMHH, 2017

Variables	Risky sexual behavior		COR [95% CI]	AOR [95% CI]
	Yes 147 (49.5%)	No 150 (51.5%)		
Sex				
Male	107 (36.0)	66 (22.2)	3.40 [2.09,5.93]***	2.23 [1.27,3.92]**
females	40 (13.5)	84 (28.3)	1	1
Age				
18–24	60 (20.2)	23 (7.7)	3.22 [2.04,5.80]***	2.08 [1.47,3.81]**
25–34	59 (19.9)	73 (24.6)	1.70 [1.03,2.73]	1.11 [0.56,1.98]
35–44	19 (6.4)	40 (13.5)	0.73 [0.16,3.40]	0.47 [0.24,0.86]
> 44	9 (3.0)	14 (4.7)	1	1
Age at onset of the disease				
≤ 25 year	93 (31.2)	75 (25.3)	1.72 [1.08–2.73]*	1.53 [0.82–2.84]
> 25 year	54 (18.2)	75 (25.3)	1	1
Type of current episode				
Manic	115 (38.7)	76 (25.6)	2.65 [1.60,4.35]***	2.31 [1.24,4.32]**
Depressive	32 (10.8)	74 (24.9)	1	1
Current alcohol use				
yes	106 (35.7)	42 (14.1)	6.64 [4.00,11.03]***	3.70 (2.01,6.78)***
No	41 (13.8)	108 (36.4)	1	1
Current khat chewing				
Yes	51 (17.2)	12 (4.0)	6.10 [3.09,12.06]***	1.48 [0.22,1.07]
No	96 (32.3)	138 (46.5)	1	1

* = *p* < 0.05, ** = *p* < 0.01, *** = *p* < 0.001, COR: crude odds ratio, AOR: adjusted odds ratio

## Discussion

Among the total participants 297 (70.0%) were recently sexually active or had sex in previous 12 months. According to a score of sexual risk indicators the overall prevalence of risky sexual behavior was 49.5, 95%CI (43.8, 55.6). This finding is higher than studies conducted in Argentina (23.9%) [22], New Zealand (16.4%) [23] and USA (26.6%) [24]. The variation could be due to differences in study design, sample size and study population in which a study in Argentina used a comparative study design involving only 60 women’s with bipolar disorder who were in a stable relationship while majority of the participants in our study were males and singles in relationship which may in turn increased RSB. Whereas, the study conducted in New Zealand included only a specific age of patients and the assessment on sexual behaviors was presented using computer with an interviewer present who could not see the subjects’ response. On the other hand, the USA finding had used a comparative study including 32 bipolar patients all subjects were literate and married. In contrary, the current finding is lower as compared with another study done in USA [25] and a study conducted in Brazil [26]. The possible reasons for this variation could be due to the fact that the USA study included higher portion of schizophrenic patients and illicit drug users in addition to bipolar patients. The inclusion of schizophrenic patients and illicit drug users in that study could increase the magnitude of RSBs because of continuous nature of schizophrenia and higher effect of illicit drugs on impairing judgment [27]. However the current finding is in line with two studies conducted in Nigeria (17) (18) and with another study done in USA [28].

In this study the odds of engaging in RSB is 2.2 times higher among males than females. Similar studies indicated that males were more likely to be involved in RSBs than females [11–13, 18]. According to the current finding the possible reasons could be substance use such as alcohol and Khat that were more commonly used among males than females. The current study found that likelihood of having RSB was higher among patients between age ranges of 18–24 than other age groups. This finding is consistent with previous study [29]. This could be explained that psychiatric disorders and RSBs both peak in young adulthood as indicated in other study findings [30, 31]. Furthermore, low self-esteem and high internal stigmatization in younger adults with mental illness may result in a failure to provide healthier romantic relationship and associated with failure to advocate for safer sex [32]. The current study also revealed that, patients who were on recent manic phase of the disorder were more likely to be engaged in RSBs as compared to those in depressive state. Previous studies also supported this finding [11, 12]. During manic episode of bipolar disorder, patients exhibit high sexual desire and frequency to engage into sexual activities which might make them more prone to exhibiting sexual risk behavior and its negative consequences. Unlike this, bipolar patients in depressive state reported loss of interest in sex [33]. Our study also indicated that substance use related problems particularly, alcohol consumption was found to increase the risk of having RSBs than those who are currently using alcohol. Many studies claim that alcohol has a disinhibiting effect and power to influence an individual’s decision making. This could lead individuals to pass wrong and unbalanced decision including entering oneself in RSBs without weighing any damage following the behavior [10, 17, 34].

In general, the present study reported the burden of RSBs and associating factors among bipolar disorder patients. However, the cross sectional nature of this study cannot allow for making cause and effect relationship between RSBs and patients characteristics. In addition, sensitive nature of sexual behaviors on face to face interview could have a social desirability bias. Moreover, this study is institution-based study. Therefore, the findings cannot be generalized to those who remain undiagnosed or untreated in the community.

## Conclusion

High prevalence of risky sexual behavior was identified among people with bipolar disorder at AMHH. Current manic episode and the consumption of alcohol were found to aggravate the risky sexual behavior. To reduce the burden of risky sexual behaviors, mental health services which focus on sexual behaviors of bipolar patients is required. Screening and intervening of risk reduction strategies for those highly vulnerable group of people should be designed. In addition, the causal mechanisms and effect between risky sexual behavior and bipolar disorder should be examined.

## Data Availability

The datasets used during the current study are available from the corresponding author on reasonable request.

## Abbreviations

AMHH: Amanuel Mental Health Hospital
AOR: Adjusted Odds Ratio
CI: Confidence Interval
COR: Crude Odds Ratio
IRB: Institution Review Board (IRB)
RSBs: Risky Sexual Behavior
USA: United States of America
